# Generalized Linear Factor Score Regression: A Comparison of Four Methods

**DOI:** 10.1177/0013164420975149

**Published:** 2020-12-11

**Authors:** Gustaf Andersson, Fan Yang-Wallentin

**Affiliations:** 1 Uppsala University, Uppsala, Sweden

**Keywords:** GLM, logistic regression, Poisson regression, meta-analysis, factor score indeterminacy, sum score methods

## Abstract

Factor score regression has recently received growing interest as an alternative for structural equation modeling. However, many applications are left without guidance because of the focus on normally distributed outcomes in the literature. We perform a simulation study to examine how a selection of factor scoring methods compare when estimating regression coefficients in generalized linear factor score regression. The current study evaluates the regression method and the correlation-preserving method as well as two sum score methods in ordinary, logistic, and Poisson factor score regression. Our results show that scoring method performance can differ notably across the considered regression models. In addition, the results indicate that the choice of scoring method can substantially influence research conclusions. The regression method generally performs the best in terms of coefficient and standard error bias, accuracy, and empirical Type I error rates. Moreover, the regression method and the correlation-preserving method mostly outperform the sum score methods.

## Introduction

In the social and psychological sciences, interest often lies in modeling relationships that involve factors. Factors are latent variables that can be inferred by the use of manifest variables, for example, questionnaire items. Common examples of factors are the Big Five personality traits, quality of life, and socioeconomic status. Structural equation modeling (SEM) is a popular tool for evaluating factor structures. The strength of SEM is its ability to consistently estimate latent variable relationships. However, because of simultaneous estimation, SEM is sensitive to bias from model misspecification and may require large samples. These drawbacks have motivated the development of alternative approaches. One such alternative is factor score regression (FSR), which has recently seen growing research interest (Hayes & Usami, 2020).

Unlike SEM, FSR is a stepwise approach to estimating factor structures. First, relationships between manifest variables and factors are estimated in a measurement model. Factors are then related to a latent or observed outcome in a regression model. Numerical values that serve as input to the regression model must be assigned to the factors. Factor score estimates are calculated for this purpose, indicating the estimated relative position of an observation on the factors (Grice, 2007). Estimated factor scores can be obtained in mainstream statistical software as they are routinely used in scale construction and as input to other statistical methods.

A sometimes overlooked fact, which is known as factor score indeterminacy, is that theoretical factor scores are not unique. In short, infinitely many sets of factor scores are consistent with a given measurement model because it is algebraically underdetermined. Various factor scoring methods, which handle the indeterminate part of factor scores differently, have been proposed. Unfortunately, recommendations on the choice of scoring method when estimating FSR models are still limited.

Another limitation of the FSR literature is the exclusive consideration of normally distributed outcomes. This is problematic because applications with other outcome types are left without theoretical guidance and justification. The generalized linear modeling framework can account for many outcome types encountered in practice. Results on the performance of scoring methods in generalized linear FSR (GLFSR) have not yet been presented in the literature.

This article compares the performance of four factor scoring methods^1^ when estimating GLFSR models. Focus is restricted to the case of normally distributed manifest variables, factor score estimates as independent variables, and an observed outcome that is either continuous (normally distributed), binary, or a count variable. In contrast to previous research, both homogeneous and heterogeneous loading structures as well as different degrees of interfactor correlation are considered. A selection of frequently applied scoring methods are evaluated,^2^ namely the regression method (Thomson, 1934; Thurstone, 1935), the correlation-preserving method by Ten Berge et al. (1999), and the total sum method and the weighted sum method described in, for example, Distefano et al. (2009). The performance of the correlation-preserving method and the sum score methods have received only limited attention in the literature on FSR methods, warranting an explicit evaluation in the current study. Evaluations are done by performing a meta-analysis on simulated data.

The rest of the article is organized as follows. First, we define the measurement model, factor scoring methods, and the regression model. Second, the simulation study design is delineated. Third, a presentation of the simulation results is provided. A discussion of the results concludes the article.

## Factor Measurement

### The Measurement Model

Confirmatory factor analysis can be used to define factors through a set of manifest variables. The measurement model is defined in this article as

(1) x=ΛF+δ,

where x is a vector of p standardized manifest variables, F is a vector of k factors, and Λ is a p×k matrix of loading coefficients. δ is assumed to be a vector of p uncorrelated measurement errors. Moreover, factors and measurement errors are independent and normally distributed such that $F\sim N_{k}(0,\Phi )$ and $\delta \sim N_{p}(0,\Theta )$ . For convenience, factor variances are fixed at unity so that Φ is a correlation matrix. Following Yang-Wallentin et al. (2010), the measurement error covariance matrix is defined as Θ=I−diag[ΛΦΛ′] when standardized manifest variables are analyzed, where I denotes the identity matrix. It follows from the measurement model set-up that $x\sim N_{p}(0,\sum =\Lambda \Phi \Lambda \prime +\Theta )$ , where ∑ is a correlation matrix. A major motivation for analyzing a completely standardized measurement model is that this enables the interpretation of each loading as the correlation coefficient between a given manifest variable and factor,^3^ assuming no cross-loadings. Because factor score indeterminacy is a function of the loadings, the restriction of each loading coefficient to the interval [−1,1] also facilitates the control of its extent.

The matrices ∑ , Λ , and Φ play a central role in factor score estimation and need to be estimated. ∑ can be estimated by the sample correlation matrix. Estimates of Λ and Φ are obtained in the estimation of Equation 1. It is well-known that using maximum likelihood (ML) estimation to apply a covariance structure to a sample correlation matrix can produce biased model parameter estimates (Cudeck, 1989). An alternative approach suggested by Jöreskog et al. (2016) is to use unweighted least squares (ULS) estimation when ∑ is a correlation matrix. It has been shown (Yang-Wallentin et al., 2010) that the ML and ULS discrepancy functions can be written on the form

(2) $$F(\Lambda ,\Phi )={\left[r-\sigma \right]}^{\prime }W^{*}\left[r-\sigma \right].$$

The vector r contains elements from the sample correlation matrix $\hat{\sum }$ . The vector σ contains elements from the population correlation matrix disregarding measurement error covariances, that is, ΛΦΛ′=∑−Θ . In ULS estimation, r and σ contain the lower diagonal elements of the corresponding matrices. Diagonal elements are included as well in ML estimation, which can produce misleading estimates because the diagonal elements of ΛΦΛ′ are not fixed at unity. The weight matrix $W^{*}$ is also defined differently in ULS and ML estimation, which influences the minimization of the discrepancy function and thus the parameter estimates. $W^{*}$ is more complex in ML estimation. To see this, let $K^{*}$ be a matrix such that left-multiplying r in ML estimation with $K^{*}$ yields r in ULS estimation. Also let $D^{*}$ be the duplication matrix and ⊗ be the Kronecker product operator. Then, $W^{*}=K^{*}(D^{*})\prime ({\hat{\sum }}^{-1}⊗{\hat{\sum }}^{-1})D^{*}(K^{*})\prime$ in ML estimation whereas $W^{*}$ is simply the identity matrix in ULS estimation.

### Factor Score Indeterminacy

The measurement model is underdetermined because the number of factors and measurement errors exceeds the number of manifest variables. To see how this affects the assignment of numerical values to factors, define the block matrix

$$\sum^{*}={\left[\begin{array}{cc} \Phi & 0\prime \\ 0 & \Theta \end{array}\right]}_{\left(p+k)\times (p+k\right)}$$

in line with Rigdon et al. (2019a), and note that

(3) $$\sum =\left[\begin{array}{cc} \Lambda & I \end{array}\right]\sum^{*}\left[\begin{array}{c} \Lambda \prime \\ I \end{array}\right].$$

When $\mathrm{rank}(\sum^{*})>\mathrm{rank}(\sum )$ , Guttman (1955) has shown that Equation 3 can be solved for F to obtain

(4) $$F=\Phi \Lambda \prime \;\sum^{-1}x+\mathrm{Bg}.$$

Factor scores in this case consist of a determinate part $\Phi \Lambda \prime \;\sum^{-1}x$ and an indeterminate part Bg , where B is a parameter matrix and g is a vector containing k orthonormal variables connected to the factors. The variables in g can be altered to generate infinitely many sets of factor scores consistent with the measurement model parameters, holding the determinate component constant, which gives rise to factor score indeterminacy.

Higher degrees of factor score indeterminacy allow for larger possible differences among sets of “true” factor scores consistent with Equation 4 (Guttman, 1955). Thus, in settings with highly indeterminate true factor scores, the validity of factor score estimation can be put into question. While factor score indeterminacy, which depends on, for example, overall loading levels (Rigdon et al., 2019a) and interfactor correlations (Rigdon et al., 2019b), does not directly influence the estimation of parameters within the measurement model, high degrees of this phenomenon translate into high degrees of indeterminacy in correlations between factors and variables outside the measurement model (Steiger, 1979). Therefore, factor score indeterminacy can severely affect conclusions about the relationship between factors and an external outcome variable.

## Factor Scoring Methods

### The Regression Method

Let E be the expectation operator. Krijnen et al. (1996) have demonstrated that the regression score estimator ${\hat{F}}_{\mathrm{reg}}$ minimizes the trace and determinant of the mean square error matrix $E[(\hat{F}-F)(\hat{F}-F)\prime ]$ . ${\hat{F}}_{\mathrm{reg}}$ is the best linear predictor of F and can be written (Skrondal & Laake, 2001) as

(5) $${\hat{F}}_{\mathrm{reg}}=\Phi \Lambda \prime \;\sum^{-1}x.$$

${\hat{F}}_{\mathrm{reg}}$ maximizes the correlation between factors and the corresponding factor score estimates, that is, $\mathrm{diag}[\mathrm{Corr}(F,{\hat{F}}_{\mathrm{reg}})]=\mathrm{diag}[\Phi \Lambda \prime \;\sum^{-1}\Lambda \Phi {]}^{\frac{1}{2}}=\rho$ . However, the covariance matrix of ${\hat{F}}_{\mathrm{reg}}$ is not equal to Φ , which can be seen through an application of the Woodbury matrix identity (Duncan, 1944). Because $\sum^{-1}=[\Lambda \Phi \Lambda \prime +\Lambda \Phi \Lambda \prime \;I(\Lambda \prime {)}^{-1}\Phi^{-1}\Lambda^{-1}\Theta {]}^{-1}$ , it follows that $E({\hat{F}}_{\mathrm{reg}}\hat{F}\prime_{\mathrm{reg}})=\Phi -\Phi (I+\Lambda \prime \Theta^{-1}\Lambda \Phi {)}^{-1}$ . Larger values of $(I+\Lambda \prime \Theta^{-1}\Lambda \Phi {)}^{-1}$ , which occur when the overall communality level increases, decrease the bias in the covariance matrix of ${\hat{F}}_{\mathrm{reg}}$ . This result should not be surprising because ${\hat{F}}_{\mathrm{reg}}$ equals only the determinate part of factor scores and the indeterminate part grows smaller when communalities increase.

Skrondal and Laake (2001) showed that the regression score estimator produces asymptotically unbiased regression coefficient estimates when the outcome variable is observed. However, Devlieger et al. (2016) found that this result is valid only when using a reference-variable approach to assign factor variance scales. The simulations by Lastovicka and Thamodaran (1991), which used the unit-variance approach, support this claim. The authors found biases in estimated regression coefficients, which grew smaller when the overall variance explained in manifest variables grew larger. Thus, ${\hat{F}}_{\mathrm{reg}}$ is expected to be biased in this article, but the bias should decrease when manifest variables have higher correlations on average with factors.

### The Correlation-Preserving Method

The correlation-preserving score estimator ${\hat{F}}_{\mathrm{corr}}$ minimizes $\det\{E[(\hat{F}-F)(\hat{F}-F)\prime ]\}$ subject to the constraint $E({\hat{F}}_{\mathrm{corr}}\hat{F}\prime_{\mathrm{corr}})=\Phi$ (Krijnen et al., 1996). Thus, ${\hat{F}}_{\mathrm{corr}}$ differs from ${\hat{F}}_{\mathrm{reg}}$ in that it is asymptotically correlation-preserving, at the cost of not maximizing its correlation with F . The correlation-preserving score estimator can be defined (Ten Berge et al., 1999) as

(6) $${\hat{F}}_{\mathrm{corr}}=\Phi^{\frac{1}{2}}{\left[\Phi^{\frac{1}{2}}\Lambda \prime \;\sum^{-1}\Lambda \Phi^{\frac{1}{2}}\right]}^{-\frac{1}{2}}\Phi^{\frac{1}{2}}\Lambda \prime \;\sum^{-1}x.$$

Other correlation-preserving score estimators exist in the literature. For example, Anderson and Rubin (1956) developed a score estimator for orthogonal factors, which was generalized to an arbitrary Φ by McDonald (1981). A comparison with Jöreskog (2000) shows that the latent variable score estimator in LISREL is equivalent to the McDonald score estimator. Moreover, this score estimator coincides with ${\hat{F}}_{\mathrm{corr}}$ when Θ is nonsingular (Ten Berge et al., 1999). Thus, ${\hat{F}}_{\mathrm{corr}}$ contains the score estimator in McDonald (1981) and LISREL as a special case.

The literature is mainly focused on ${\hat{F}}_{\mathrm{corr}}$ with $E({\hat{F}}_{\mathrm{corr}}\hat{F}\prime_{\mathrm{corr}})=I$ , that is, the Anderson-Rubin score method. Lastovicka and Thamodaran (1991) found that the Anderson-Rubin method is associated with less biased coefficient estimates than the regression method in FSR, including situations with correlated factors. However, it is still unknown how the correlation-preserving method and the regression method compare when ${\hat{F}}_{\mathrm{corr}}$ is not restricted to produce factor score estimates that asymptotically reflect uncorrelated factors.

### Sum Score Methods

Sum score methods have been understudied in the FSR literature, partly because these methods are less sophisticated approaches and are expected to have poorer performance than other scoring methods. Sum scoring is regularly encountered in practice because of its simplicity, which motivates an explicit comparison of this class of factor scoring methods together with more sophisticated approaches.

The total sum method and the weighted sum method are considered in this article. The total sum score estimator is defined as

(7) $${\hat{F}}_{\mathrm{tot}}=(\Lambda^{*})\prime x.$$

$\Lambda^{*}$ equals the loading matrix Λ after nonzero elements have been replaced with either −1 or 1 depending on their original signs (Beauducel & Hilger, 2019). If all manifest variables have the same polarity, then the estimated factor score for an observation on a given factor is simply the sum of observed values on the manifest variables correlated with the factor.

A drawback of ${\hat{F}}_{\mathrm{tot}}$ is that all relevant manifest variables have equal weights. The weighted sum method ${\hat{F}}_{\mathrm{wei}}$ addresses this issue by considering Λ instead of $\Lambda^{*}$ (Distefano et al., 2009). Weights then depend on the strength of linear relationship between manifest variables and factors. ${\hat{F}}_{\mathrm{wei}}$ is defined as

(8) $${\hat{F}}_{\mathrm{wei}}=\Lambda \prime \;x.$$

Note that one expects the performance of ${\hat{F}}_{\mathrm{wei}}$ to approach that of ${\hat{F}}_{\mathrm{tot}}$ when communalities grow larger. In comparison with the regression method and the correlation-preserving method, ${\hat{F}}_{\mathrm{tot}}$ and ${\hat{F}}_{\mathrm{wei}}$ do not maximize correlations with F nor preserve interfactor correlations.

## The Regression Model

Suppose that a set of factors F is related to an n×1 outcome vector y by the k×1 vector of regression weights γ . n here denotes the sample size. The n×k matrix of factor score estimates $\hat{F}$ replaces F in GLFSR because F is unobserved. $\hat{F}$ is related to y by the k×1 vector of regression weights $\gamma^{*}$ , whose elements depend on which factor scoring method is used to calculate $\hat{F}$ . Defining the conditional outcome $E(y|\hat{F})\equiv \mu_{y}$ , the GLFSR model can be written^4^ as

(9) $$g(\mu_{y})=\hat{F}\gamma^{*}.$$

The link function g enables the modeling of a variety of outcome distributions. Conditional on the explanatory variables, each outcome realization is assumed to be independent and from an exponential dispersion family distribution (Agresti, 2015). Ordinary FSR based on normally distributed outcomes is a special case of GLFSR, where the identity link $g(\mu_{y})=\mu_{y}$ is applied (Madsen & Thyregod, 2010). Other common link functions are the logit link $g(\mu_{y})=\log\left[\mu_{y}/(1-\mu_{y})\right]$ for logistic regression and the log link $g(\mu_{y})=\log\mu_{y}$ for Poisson regression.

Maximum likelihood estimation can be applied to estimate $\gamma^{*}$ . Let $L(\gamma^{*})$ be the likelihood function and define $l(\gamma^{*})=\log L(\gamma^{*})$ . The maximum likelihood estimator maximizes $l(\gamma^{*})$ or equivalently $L(\gamma^{*})$ . This occurs when ${\hat{\gamma }}^{*}$ is the solution (Agresti, 2015) to

(10) $$\frac{\partial l(\gamma^{*})}{\partial \gamma^{*}}=\hat{F}\prime \;DV^{-1}(y-\mu_{y})=0,$$

where $D=\mathrm{diag}[\frac{\partial \mu_{1}}{\partial \eta_{1}},\frac{\partial \mu_{2}}{\partial \eta_{2}},\ldots ,\frac{\partial \mu_{n}}{\partial \eta_{n}}]$ and $V=\mathrm{diag}[\mathrm{Var}(y_{1}),\mathrm{Var}(y_{2}),\ldots ,\mathrm{Var}(y_{n})]$ . ${\hat{\gamma }}^{*}$ is found by iterative algorithms such as the Newton-Raphson or Fisher scoring method because Equation 11 generally has no closed-form solution. The Fisher scoring method uses the expected information matrix $I(\gamma^{*})$ to update the parameter estimator until convergence is reached, that is, when the difference between the current and previous update is sufficiently small (Stroup, 2013). Under regularity conditions,^5^ it has been shown (Madsen & Thyregod, 2010) that

$${\hat{\gamma }}^{*}-\gamma^{*}\overset{d}{\to }Z\sim N\left(0,I^{-1}(\gamma^{*})\right).$$

Thus, ${\hat{\gamma }}^{*}$ is an asymptotically biased estimator of γ when $\gamma^{*}\neq \gamma$ . However, the practical importance of finite-sample biases in coefficient and standard error estimates has yet to be examined in GLFSR.

## Study Design

### Simulation Parameters

A simulation study was performed to compare the factor scoring methods in GLFSR. The simulation parameters are presented in Table 1. A total of 1,000 replications were made for each specific condition. A total of 3,780 conditions were considered in this article.

**Table 1.** Simulation Parameters.

Parameter	Value
Mean of loading distribution $\left(\mu_{\lambda }\right)$	0.40, 0.60, 0.80
Standard deviation of loading distribution $\left(\sigma_{\lambda }\right)$	0.00, 0.03, 0.09
Inter-factor correlation $\left(\phi_{12}\right)$	0.00, ±0.30, ±0.50, ±0.80
Sample size (n)	50, 100, 200, 500, 1,000
Factor scoring method	regression, correlation-preserving, total sum, weighted sum
Regression model	ordinary, logistic, Poisson
Number of replications per condition	1,000

#### The Loading-Generating Distribution

Loading coefficients are often assumed to be equal in the literature. This assumption is unrealistic in many research settings. We adopted a new approach to defining the loading-generating distribution. Loading coefficients were in each replication drawn from a uniform distribution with endpoints depending on a condition-specific mean and standard deviation.^6^ Loading coefficients were ensured to fall into a limited section of the interval [0.244,0.956] . We thus eliminated the issues of drawing unequally likely or impossible loadings, which were prevalent in some previous studies. Mean loading levels were selected from Koran (2020), ranging from relatively low $\left(\mu_{\lambda }=0.40\right)$ to relatively high $\left(\mu_{\lambda }=0.80\right)$ . Both equal $\left(\sigma_{\lambda }=0\right)$ and unequal $\left(\sigma_{\lambda }>0\right)$ loading coefficients were considered.

#### Interfactor Correlation

A two-factor measurement model, which is the simplest multifactor measurement model, was assumed. We considered both uncorrelated $\left(\phi_{12}=0\right)$ and correlated $\left(\phi_{12}\neq 0\right)$ factors.

#### Sample Size

Fifty, 100, 200, 500, and 1,000 observations were analyzed as in Devlieger et al. (2019), reflecting common sample sizes in structural equation modeling.

#### Factor Scoring Method and Regression Model

The factor scoring methods mentioned in the Introduction were considered. The GLFSR models in the simulation study corresponded to Equation 10. The models had an observed outcome and factor score estimates, based on factors with normally distributed manifest variables, as independent variables.

### Data-Generating Process

**Figure 1.**
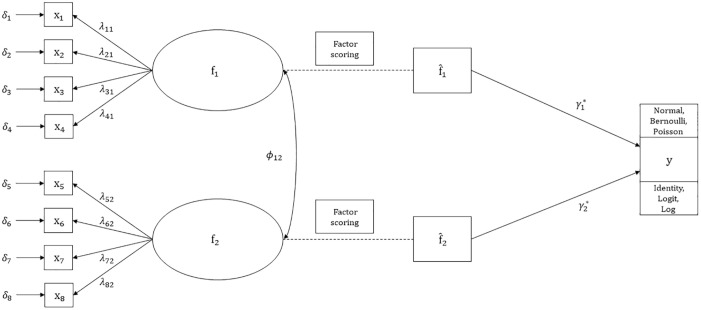
Path diagram of simulation model.

Figure 1 displays a path diagram of the simulation measurement model. Two factors, $f_{1}$ and $f_{2}$ , were analyzed. The first four out of eight manifest variables $\left(x_{1},x_{2},\ldots ,x_{8}\right)$ were related to $f_{1}$ and the rest to $f_{2}$ by the loading coefficients $\left(\lambda_{11},\lambda_{21},\lambda_{31},\lambda_{41}\right)$ and $\left(\lambda_{52},\lambda_{62},\lambda_{72},\lambda_{82}\right)$ . Factors were related by the interfactor correlation coefficient $\phi_{12}$ .

For each unique combination of the simulation parameter values, data were generated in the following steps in R (R Development Core Team, 2019):

1. True factor scores were generated by drawing n observations from a $N_{2}(0,\Phi )$ distribution. Loading coefficients were sampled from a U(a,b) distribution. Manifest variable data were generated as linear combinations of Λ , F , and δ in line with Equation 1. Free measurement model parameters were estimated using unweighted least squares estimation with robust standard errors in the lavaan package (Rosseel, 2012).
2. Factor score estimates were calculated. True factor scores were used to generate outcome values, where the true regression model had coefficients γ=(1,2) . γ was estimated with all combinations of regression model and factor scoring method using base R. Last, nonconvergence information as well as coefficient and standard error estimates were extracted.

The inverse link of the linear predictor was used to generate outcome values (Carsey & Harden, 2013). Let $f_{1i}$ and $f_{2i}$ , i=1,2,…,n , be individual factor scores on the first and second factor. A conditional variance of 10 for each outcome realization $y_{i}$ was arbitrarily selected such that $y_{i}|f_{1i},f_{2i}\sim N(f_{1i}+2f_{2i},10)$ in ordinary FSR. In logistic FSR, each $y_{i}$ was conditionally Bernoulli-distributed such that $y_{i}|f_{1i},f_{2i}\sim \mathrm{Be}[\exp(f_{1i}+2f_{2i})/\left(1+\exp(f_{1i}+2f_{2i})\right)]$ . In Poisson FSR, each $y_{i}$ was conditionally Poisson-distributed such that $y_{i}|f_{1i},f_{2i}\sim \mathrm{Poi}[\exp(f_{1i}+2f_{2i})]$ .

### Performance Assessment

Data were evaluated by the use of five performance criteria listed in Table 2. There were no evident reasons for results to differ between the factors in Figure 1. Thus, focus was restricted to the first factor. Data were trimmed by discarding cases with the upper and lower 0.50 percent of ${\hat{\gamma }}_{1}^{*}$ values in each unique condition to reduce the negative influence of outliers.

**Table 2.** Performance Criteria.

Criterion	Definition
Nonconvergence rate (%)	$\frac{100}{C_{\mathrm{tot}}}\;\sum_{c=1}^{C_{\mathrm{tot}}}\;I(A_{c})$
Relative bias (%)	$\mathrm{RB}=\frac{100}{C}\;\sum_{c=1}^{C}\;({\hat{\gamma }}_{c}-1)$
Relative standard error bias (%)	$\mathrm{RSEB}=100\left(\frac{\frac{1}{C}\;\sum_{c=1}^{C}\;\hat{S}E_{c}}{\sqrt{\frac{1}{C-1}\;\sum_{c=1}^{C}\;{\left({\hat{\gamma }}_{c}-\bar{\gamma }\right)}^{2}}}-1\right)$
Root mean square error	$\mathrm{RMSE}=\sqrt{\frac{1}{C}\;\sum_{c=1}^{C}\;{\left({\hat{\gamma }}_{c}-1\right)}^{2}}$
Empirical Type I error rate (%)	$\hat{\alpha }=\frac{100}{C}\;\sum_{c=1}^{C}\;I({\hat{p}}_{c}<0.05)$

*Note*. $C_{\mathrm{tot}}$ = replications per condition. C = convergent replications per condition after data trimming. $\hat{\alpha }$ = empirical Type I error rate.

Given the recommendations by Skrondal (2000), meta-analysis models were formulated to relate the simulation parameters in Table 1 to the criteria. Separate analyses of variance (ANOVA) with first-order interactions were performed in line with Devlieger et al. (2016). In contrast to the majority of previous FSR studies, we examined practical significance in terms of standardized effect size as preferred by Boomsma (2013).

First, Type III sum of squares decompositions were evaluated in terms of effect size. Second, the most informative relationships were analyzed in relation to established guidelines. The partial omega-squared ${\hat{\omega }}_{p}^{2}$ was selected as effect size measure because it is a less biased, population-based, version of the conventional partial eta-squared (Lakens, 2013). ${\hat{\omega }}_{p}^{2}$ can be interpreted as the estimated proportion of the total variance in the dependent variable explained by a given independent variable when the influence of other independent variables has been eliminated. ${\hat{\omega }}_{p}^{2}$ for a given independent variable is defined in a fixed-effects design as

(11) $${\hat{\omega }}_{p}^{2}=\frac{df_{\mathrm{effect}}(\mathrm{MS}S_{\mathrm{effect}}-\mathrm{MS}S_{\mathrm{error}})}{df_{\mathrm{effect}}\mathrm{MS}S_{\mathrm{effect}}+(N-df_{\mathrm{effect}})\mathrm{MS}S_{\mathrm{error}}},$$

where $df_{\mathrm{effect}}$ denotes the degrees of freedom, $\mathrm{MS}S_{\mathrm{effect}}$ is the mean sum of squares for the independent variable, N=3,780 is the number of observations, and $\mathrm{MS}S_{\mathrm{error}}$ is the mean error sum of squares (Olejnik & Algina, 2003). Bootstrapped 95% confidence intervals were reported for ${\hat{\omega }}_{p}^{2}$ . In lack of a systematic evaluation of effect sizes in previous FSR research, confidence intervals for ${\hat{\omega }}_{p}^{2}$ that covered any value in the interval [0.14,1.00] were deemed to signify practically important effects. ${\hat{\omega }}_{p}^{2}>0.14$ suggests a large effect according to Cohen (2013).

#### Nonconvergence Rates

The indicator function $I(A_{c})$ equaled 1 if estimates of ∑ , Φ , or Θ were not positive definite, if $|\phi_{12}|>1$ , if loading estimates were missing, or if regression coefficient and standard error estimates were missing. We expected nonconvergence rates to be lower when more observations were analyzed, manifest variables had stronger correlations on average with factors, and factors were weakly correlated. In addition, factor scoring methods were expected to have approximately the same proportion of nonconvergent replications based on the findings by Devlieger et al. (2016). Nonconvergence rates exceeding 10% were considered problematic in this article.

#### Bias

The relative bias compares estimated regression coefficients with the true coefficient value 1. The relative standard error bias compares the mean estimated standard error with the empirical standard deviation acting as an estimate of the theoretical standard error. Regression coefficients and standard errors were expected to mostly be underestimated based on previous simulation studies, for example, Skrondal and Laake (2001) and Devlieger et al. (2016). Moreover, biases were expected to generally decrease when the mean loading level grew higher. The sum score methods were expected to mainly be associated with substantial biases because they utilize little model information. We also expected bias levels for the regression method and the correlation-preserving method to converge and approach zero for higher mean loading levels. Following Hoogland and Boomsma (1998), absolute values of coefficient and standard error biases less than 5% and 10% were considered acceptable.

#### Root Mean Square Error (RMSE)

The RMSE can be considered an indicator of accuracy because it depends on both estimator bias and variance. A lower RMSE indicates higher accuracy. We expected higher accuracy for higher mean loading levels. In addition, RMSE values for the regression method and the correlation-preserving method were expected to decrease when analyzing larger samples.

#### Empirical Type I Error Rates $\left(\hat{\alpha }\right)$

The indicator function $I({\hat{p}}_{c}<0.05)$ equaled 1 if the estimated *p* value for a two-sided *t* test of $H_{0}:\gamma_{1}^{*}=1$ was less than 5%. Pronounced over-rejections were expected to occur, for example, when manifest variables were weakly correlated with factors. Furthermore, we expected the increased rejection power from larger samples to produce higher $\hat{\alpha }$ levels when biases were present. Empirical Type I error rates outside of the interval [0.025,0.075] were deemed problematic in line with the “liberal” criterion suggested by Bradley (1978).

## Results

Simulations resulted in 3,780,000 replications, of which about 3.60% were nonconvergent. A total of 3,606,180 replications were available for meta-analysis after the data trimming described in the previous section. Table 3 presents descriptive statistics for the simulated data.

**Table 3.** Descriptive Statistics by Regression Model and Factor Scoring Method.

		Mean				*SD*				Min				Max			
		Reg.	Corr.	Tot.	Wei.	Reg.	Corr.	Tot.	Wei.	Reg.	Corr.	Tot.	Wei.	Reg.	Corr.	Tot.	Wei.
Ordinary	Non-conv. (%)	3.60	3.60	3.60	3.60	7.85	7.85	7.85	7.85	0.00	0.00	0.00	0.00	37.00	37.00	37.00	37.00
	RB (%)	−5.24	−24.27	−74.08	−56.11	12.61	23.22	13.35	28.15	−55.06	−94.58	−107.59	−114.49	52.10	13.16	−48.10	25.11
	RSEB (%)	−3.04	−5.20	−0.23	−5.65	10.30	9.14	4.46	10.16	−40.00	−39.01	−27.56	−42.73	17.36	13.01	9.35	13.89
	RMSE	0.60	0.49	0.75	0.64	0.53	0.29	0.13	0.22	0.10	0.11	0.50	0.13	2.71	1.48	1.09	1.21
	$\hat{\alpha }$ (%)	6.94	22.95	98.37	74.74	4.09	24.59	5.75	31.96	2.15	2.38	53.65	6.40	22.61	98.8	100.00	100.00
Poisson	Non-conv. (%)	3.60	3.60	3.60	3.60	7.85	7.85	7.85	7.85	0.00	0.00	0.00	0.00	37.00	37.00	37.00	37.00
	RB (%)	−10.13	−27.94	−75.08	−58.36	14.06	22.34	12.54	25.79	−56.66	−91.14	−105.74	−113.91	39.50	8.39	−51.82	16.86
	RSEB (%)	−86.72	−86.76	−85.49	−85.70	9.11	10.12	12.14	12.12	−97.89	−98.49	−98.89	−98.68	−54.88	−48.57	−46.02	−45.24
	RMSE	0.59	0.51	0.76	0.67	0.52	0.28	0.12	0.18	0.09	0.13	0.55	0.28	2.61	1.49	1.06	1.14
	$\hat{\alpha }$ (%)	80.72	90.18	99.92	98.61	12.37	8.00	0.22	2.10	42.13	56.86	98.44	91.55	96.79	100.00	100.00	100.00
Logistic	Non-conv. (%)	3.60	3.60	3.60	3.60	7.85	7.85	7.85	7.85	0.00	0.00	0.00	0.00	37.00	37.00	37.00	37.00
	RB (%)	−20.56	−36.61	−78.48	−64.82	15.32	23.00	10.63	19.49	−64.22	−96.17	−106.21	−111.86	31.79	15.07	−59.76	−17.42
	RSEB (%)	−10.77	−9.74	−2.98	−8.78	12.83	11.08	6.03	9.74	−55.03	−52.67	−24.90	−43.96	6.59	3.94	9.84	5.14
	RMSE	0.56	0.52	0.79	0.68	0.43	0.24	0.10	0.18	0.13	0.14	0.64	0.20	2.26	1.24	1.07	1.15
	$\hat{\alpha }$ (%)	21.45	46.60	98.90	87.47	16.46	33.60	3.29	20.11	1.84	2.64	78.60	20.76	80.70	100.00	100.00	100.00

*Note*. Reg. = regression; Corr. = correlation-preserving; Tot. = total sum; Wei. = weighted sum; Non-conv. = nonconvergence; RB = relative bias; RSEB = relative standard error bias; RMSE = root mean square error. $\hat{\alpha }$ = empirical Type I error rate.

All regression models were possible to estimate given a properly estimated measurement model. This is in line with the fact that nonconvergence statistics were equal for all regression models and factor scoring methods. Coefficients and standard errors were indicated to be generally underestimated. Root mean square error statistics were similar across the regression models, indicating similar accuracy in ordinary, logistic, and Poisson FSR. In addition, mean empirical Type I error rates generally fell outside of the acceptance interval, suggesting pronounced null hypothesis over-rejections in many cases.

Table 4 displays sum of squares decompositions for the meta-analyses. The volatility of loading coefficients $\left(\sigma_{\lambda }\right)$ had a limited impact on the performance criteria. Estimated location effects^7^ for $\sigma_{\lambda }$ were marginal, indicating no notable difference in performance criteria when equal or unequal loading coefficients were considered.

**Table 4.** Sum of Squares Decompositions.

	Degrees of freedom	Non-conv.		RB		RSEB		RMSE		$\hat{\alpha }$	
		Sum of squares	${\hat{\omega }}_{p}$	Sum of squares	${\hat{\omega }}_{p}$	Sum of squares	${\hat{\omega }}_{p}$	Sum of squares	${\hat{\omega }}_{p}$	Sum of squares	${\hat{\omega }}_{p}$
Intercept	1	15,825		67,299		2,309		16.251		9,651	
$\mu_{\lambda }$	2	27,530	[0.809, 0.825]	72,362	[0.234, 0.279]	12,895	[0.116, 0.155]	5.032	[0.080, 0.115]	16,724	[0.032, 0.057]
$\sigma_{\lambda }$	2	340	[0.039, 0.066]	32	[−, 0.001]	59	[−, 0.003]	0.000	[−, −]	22	[−, 0.001]
$\phi_{12}$	6	4,246	[0.386, 0.429]	129,218	[0.358, 0.402]	1,415	[0.009, 0.024]	14.880	[0.218, 0.263]	64,749	[0.130, 0.171]
*n*	4	24,423	[0.790, 0.808]	12,828	[0.043, 0.072]	900	[0.005, 0.017]	9.271	[0.144, 0.186]	39,154	[0.079, 0.114]
Method	3	0	[−, −]	63,306	[0.209, 0.254]	3,846	[0.032, 0.058]	5.151	[0.081, 0.117]	209,429	[0.342, 0.387]
Link	2	0	[−, −]	9,013	[0.029, 0.054]	27,3851	[0.758, 0.779]	0.004	[−, 0.001]	209,517	[0.343, 0.387]
$\mu_{\lambda }$ : $\sigma_{\lambda }$	4	175	[0.018, 0.038]	172	[−, 0.002]	96	[−, −]	0.004	[−, −]	88	[−, 0.001]
$\mu_{\lambda }$ : $\phi_{12}$	12	8,287	[0.556, 0.591]	33,999	[0.118, 0.157]	4,487	[0.036, 0.063]	12.259	[0.184, 0.227]	27,076	[0.052, 0.083]
$\mu_{\lambda }$ : *n*	8	75,859	[0.921, 0.928]	31,189	[0.109, 0.148]	7,167	[0.063, 0.095]	6.591	[0.103, 0.141]	11,620	[0.019, 0.041]
$\mu_{\lambda }$ : Method	6	0	[−, −]	233,438	[0.507, 0.545]	19,832	[0.172, 0.275]	39.076	[0.433, 0.475]	262,108	[0.396, 0.439]
$\mu_{\lambda }$ : Link	4	0	[−, −]	18,891	[0.066, 0.099]	7,402	[0.066, 0.099]	0.776	[0.009, 0.024]	35,856	[0.073, 0.106]
$\sigma_{\lambda }$ : $\phi_{12}$	12	72	[0.003, 0.016]	161	[−, −]	393	[−, 0.007]	0.101	[−, 0.002]	72	[−, −]
$\sigma_{\lambda }$ : *n*	8	866	[0.103, 0.142]	146	[−, 0.000]	3,086	[0.024, 0.047]	0.022	[−, −]	46	[−, −]
$\sigma_{\lambda }$ : Method	6	0	[−, −]	272	[−, 0.003]	1,720	[0.011, 0.029]	0.073	[−, 0.003]	217	[−, 0.001]
$\sigma_{\lambda }$ : Link	4	0	[−, −]	62	[−, 0.001]	1,911	[0.014, 0.032]	0.007	[−, 0.000]	70	[−, 0.000]
$\phi_{12}$ : *n*	24	2,538	[0.266, 0.312]	2,898	[0.002, 0.015]	1,771	[0.008, 0.025]	6.076	[0.092, 0.129]	481	[−, −]
$\phi_{12}$ : Method	18	0	[−, −]	288,663	[0.560, 0.595]	17,158	[0.148, 0.190]	46.544	[0.477, 0.516]	91,302	[0.176, 0.219]
$\phi_{12}$ : Link	12	0	[−, −]	8,859	[0.027, 0.051]	82,629	[0.480, 0.519]	3.035	[0.044, 0.073]	85,733	[0.167, 0.210]
*n*: Method	8	0	[−, −]	13,146	[0.043, 0.071]	3,073	[0.023, 0.045]	33.882	[0.397, 0.440]	64,186	[0.128, 0.168]
*n*: Link	8	0	[−, −]	4,938	[0.013, 0.031]	19,622	[0.170, 0.213]	0.951	[0.010, 0.028]	28,999	[0.057, 0.088]
Method: Link	6	0	[−, −]	10,805	[0.035, 0.062]	6,064	[0.053, 0.083]	0.708	[0.007, 0.022]	641,790	[0.622, 0.653]
Residuals	3,616	5,880		200,903		78,825		44.845		348,481	

*Note*. Negative 95% confidence interval endpoints for ${\hat{\omega }}_{p}^{2}$ have been replaced with “−”. Non-conv. = nonconvergence; RB = relative bias; RSEB = relative standard error bias; RMSE = root mean square error. $\hat{\alpha }$ = empirical Type I error rate.

### Nonconvergence Rates

**Figure 2.**
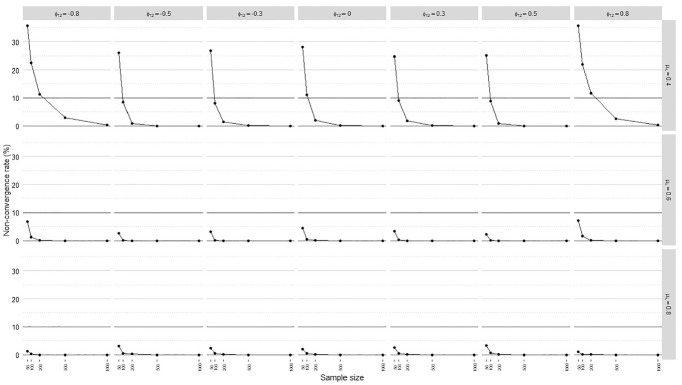
Nonconvergence rates.

Only simulation parameters related to the measurement model influenced nonconvergence rates according to the meta-analysis. Nonconvergence rates averaged over loading volatility levels, regression models, and factor scoring methods are displayed in Figure 2, including a horizontal line marking the 10% acceptance threshold.

The meta-analysis in Table 4 suggested an important interaction between the mean loading level, sample size, and interfactor correlation. In particular, sample size and interfactor correlation had notable impacts only when manifest variables were weakly correlated with factors on average $\left(\mu_{\lambda }=0.40\right)$ . Nonconvergence rates were less than 10% in all cases when manifest variables had relatively high mean correlations with factors $\left(\mu_{\lambda }=[0.60,0.80]\right)$ .

Nonconvergence rates were the most problematic when manifest variables on average weakly indicated the factors $\left(\mu_{\lambda }=0.40\right)$ and factors were highly correlated $\left(|\phi_{12}|=0.80\right)$ . In these cases, at least 500 observations were needed to keep nonconvergence rates below 10%. Samples of at least 100 observations mostly sufficed when $\mu_{\lambda }=0.40$ and $|\phi_{12}|<0.80$ .

### Relative Bias

**Figure 3.**
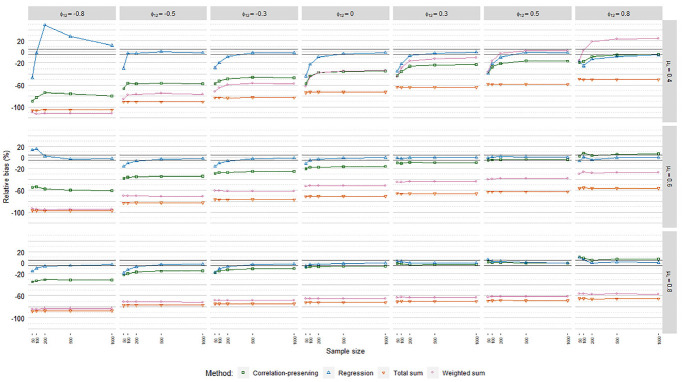
Relative coefficient bias, ordinary factor score regression.

**Figure 4.**
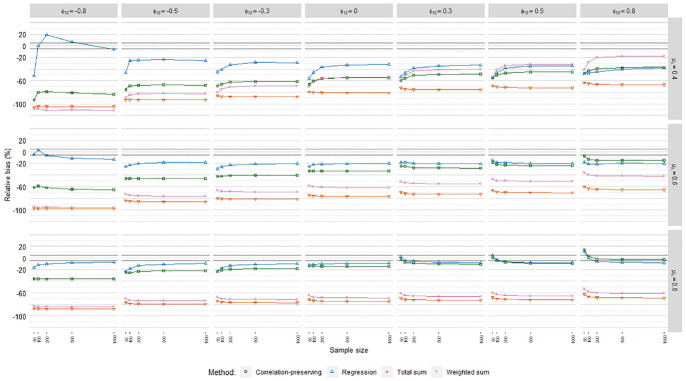
Relative coefficient bias, logistic factor score regression.

**Figure 5.**
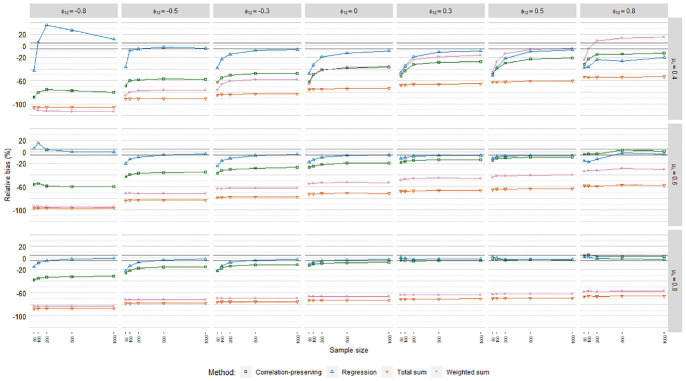
Relative coefficient bias, Poisson factor score regression.

The meta-analysis in Table 4 suggested similar relative bias (RB) levels across the regression models. However, comparing logistic and Poisson to ordinary FSR, RB levels were in some cases shifted downward to an extent that influenced bias acceptability. RB values averaged over loading volatility levels are thus displayed for each regression model in Figures 3, 4, and 5, including 5% acceptance intervals.

In line with our expectations, coefficients were generally underestimated. The mean loading level had an impact on biases according to the meta-analysis in Table 4, but it depended on the specific factor scoring method. RB values for the regression method and the correlation-preserving method converged and approached zero for higher mean loading levels. In contrast, bias decreases were marginal for the total sum method. RB values for the weighted sum method in fact increased for higher mean loading levels. Biases were almost always severe for the sum score methods, which therefore were discarded from further RB analysis.

Coefficients were often less biased with the regression method than the correlation-preserving method. Compared with $\phi_{12}=0$ , RB levels for the correlation-preserving method shifted downward when $\phi_{12}<0$ and upward when $\phi_{12}>0$ . Because RB values for the regression method did not follow this pattern, considerable differences between the two methods arose when factors were negatively correlated.

The correlation-preserving method was mostly associated with unacceptable RB levels that were less severe when manifest variables had relatively high mean correlations with factors $\left(\mu_{\lambda }=[0.60,0.80]\right)$ and factors were positively correlated. In ordinary FSR, the regression method performed acceptably in most cases when at least 500 observations were analyzed. In addition, smaller sample sizes could often provide acceptable RB levels for the regression method when there was a relatively high mean loading level $\left(\mu_{\lambda }=[0.60,0.80]\right)$ and $\phi_{12}>0$ . In logistic FSR, no factor scoring method had a consistently acceptable bias level. In Poisson FSR, RB values for the regression method were within or close to the edges of the acceptance interval when at least 500 observations were considered and manifest variables had relatively high mean correlations with factors $\left(\mu_{\lambda }=[0.60,0.80]\right)$ .

### Relative Standard Error Bias

**Figure 6.**
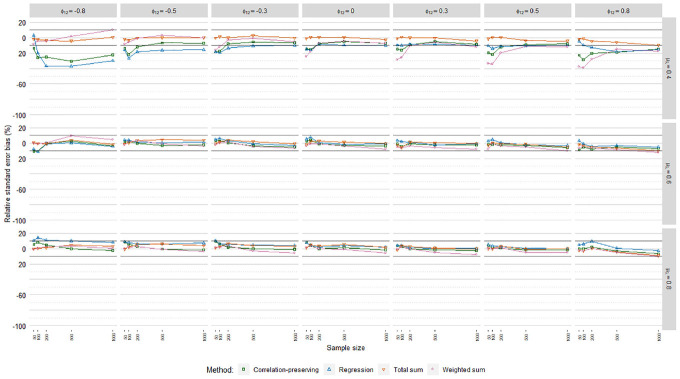
Relative standard error bias, ordinary factor score regression.

**Figure 7.**
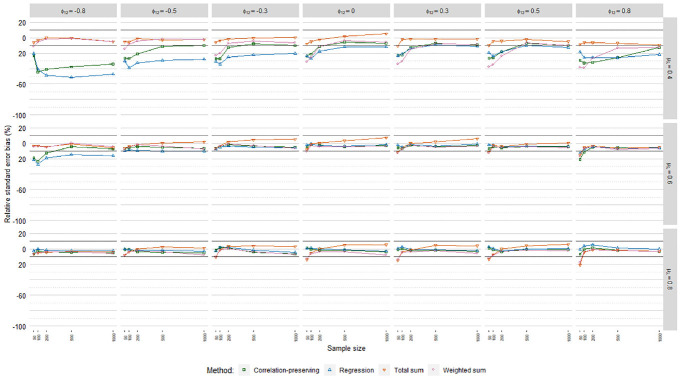
Relative standard error bias, logistic factor score regression.

**Figure 8.**
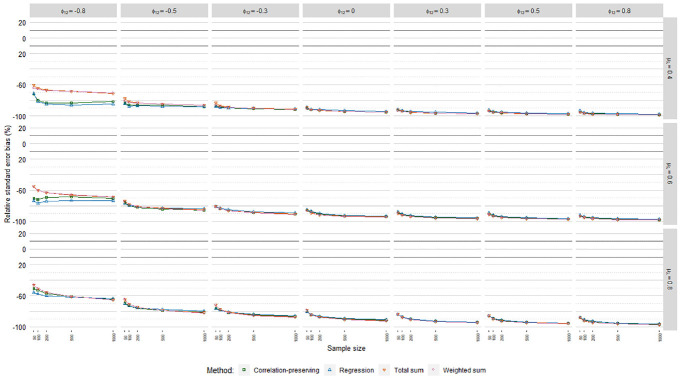
Relative standard error bias, Poisson factor score regression.

Figures 6, 7, and 8 display relative standard error bias (RSEB) values averaged over levels of loading variance for each regression model, including 10% acceptance intervals. The meta-analysis in Table 4 suggested that substantial RSEB differences exist across regression models. Estimated standard errors were severely biased in all cases in Poisson FSR. RSEB values were mostly less than −60% in this case. In contrast, absolute RSEB values were mainly acceptable in ordinary and logistic FSR. For these two regression models, problematic RSEB values were found primarily in situations where manifest variables were weakly correlated with factors on average $\left(\mu_{\lambda }=0.40\right)$ and factors were correlated. Contrary to our expectations, no substantial differences in RSEB values were found between the sum score methods and the other methods.

### Root Mean Square Error

**Figure 9.**
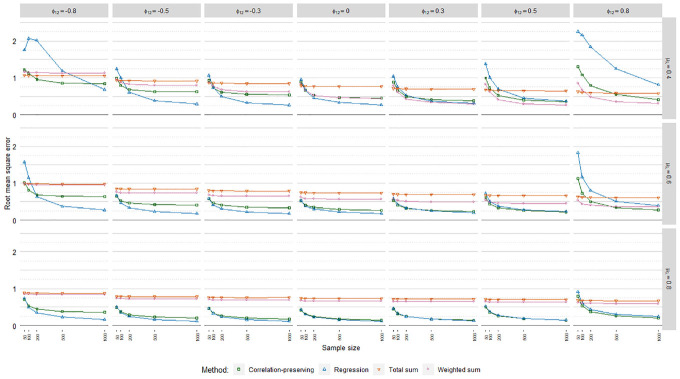
Root mean square error.

The meta-analysis in Table 4 on root mean square error (RMSE) values indicated no noteworthy differences across the regression models. RMSE values averaged over loading volatility levels and regression models are displayed in Figure 9. As expected, the regression method and the correlation-preserving method displayed higher accuracy in larger samples. In contrast, the sum score methods were, in large, unaffected by sample size.

The regression method and the correlation-preserving method mostly outperformed the sum score methods in terms of accuracy. In addition, the regression method generally had the highest accuracy. The accuracy of the regression method and the correlation-preserving method increased when the mean loading level grew larger and factors were weakly correlated. Moreover, the negative influence of interfactor correlations on the accuracy of the regression method and the correlation-preserving method decreased notably when the mean loading level increased. Accuracy differences between the regression method and the correlation-preserving method were, in large, negligible when manifest variables had relatively high mean correlations with factors $\left(\mu_{\lambda }=[0.60,0.80]\right)$ and factors were positively correlated.

### Empirical Type I Error Rates

**Figure 10.**
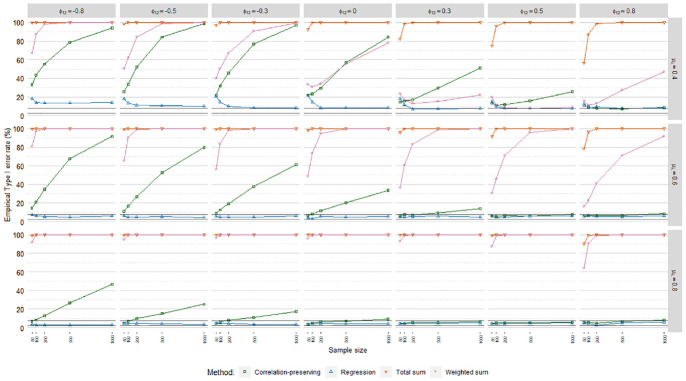
Type I error rates, ordinary factor score regression.

**Figure 11.**
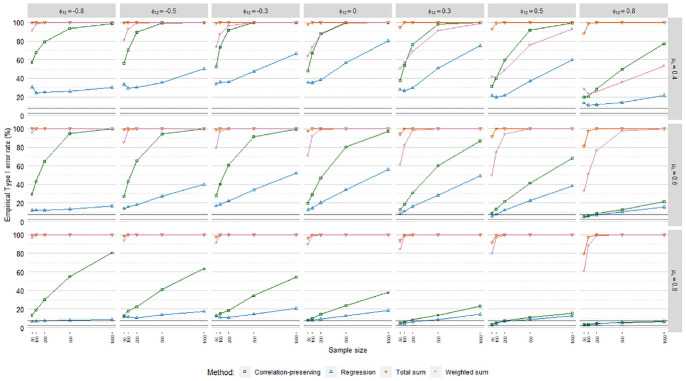
Type I error rates, logistic factor score regression.

**Figure 12.**
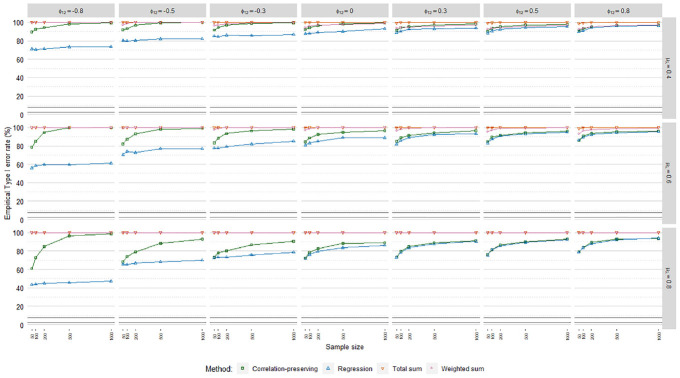
Type I error rates, Poisson factor score regression.

Empirical Type I error rates $\left(\hat{\alpha }\right)$ averaged over loading volatility levels are displayed in Figures 10, 11, and 12, including acceptance intervals covering values from 2.50% to 7.50%. The meta-analysis in Table 4 suggested differences in $\hat{\alpha }$ patterns across the regression models. Poisson FSR stood out with severely inflated Type I error rates in all cases, caused in part by severe standard error underestimations.

The sum score methods had more problematic $\hat{\alpha }$ patterns than the other methods. For instance, $\hat{\alpha }$ values for the total sum method were almost always at 100%. The sum score methods were discarded from further $\hat{\alpha }$ analysis due to their problematic performance.

The meta-analysis in Table 4 indicated a noteworthy impact of the mean loading level on empirical Type I error rates. This was reflected in $\hat{\alpha }$ levels for the regression method and the correlation-preserving method in ordinary and logistic FSR, where over-rejections were the most severe when manifest variables were on average weakly correlated with factors. Moreover, in line with the relative bias results, $\hat{\alpha }$ levels were more problematic for the correlation-preserving method when factors were negatively correlated. The meta-analysis also confirmed the expected impact of sample size on empirical Type I error rates. Focusing on ordinary and logistic FSR, larger samples were associated with higher $\hat{\alpha }$ values for the regression method and the correlation-preserving method when coefficient biases were present. Increases in $\hat{\alpha }$ levels when analyzing larger samples were greater for the correlation-preserving method than the regression method.

$\hat{\alpha }$ levels in logistic FSR were mainly unacceptable. However, in ordinary FSR, the regression method mostly displayed acceptable $\hat{\alpha }$ levels when manifest variables had relatively high mean correlations with factors $\left(\mu_{\lambda }=[0.60,0.80]\right)$ . In contrast, the correlation-preserving method had unacceptable $\hat{\alpha }$ levels in the majority of cases. As expected from the relative bias results, differences in $\hat{\alpha }$ levels between the regression method and the correlation-preserving method were negligible when the mean loading level was relatively high $\left(\mu_{\lambda }=[0.60,0.80]\right)$ and $\phi_{12}>0$ .

## Discussion

In this article, we examined the performance of a selection of factor scoring methods when estimating regression coefficients in GLFSR. Our simulations indicate that relationships between factors and an observed outcome are recovered with approximately the same accuracy in ordinary, logistic, and Poisson FSR. However, levels of coefficient and standard error bias associated with each factor scoring method differed substantially across regression models in some cases. Therefore, we conclude that ordinary FSR results are not representative for GLFSR as a whole.

### Ordinary Factor Score Regression

The results indicate that the regression method has the best performance when analyzing a normally distributed outcome. Given sufficiently large samples, the regression method was associated with mostly acceptable levels of coefficient bias and empirical Type I errors unlike the other factor scoring methods. The lesser bias of the correlation-preserving method in Lastovicka and Thamodaran (1991), compared with the regression method, was consequently not confirmed in most cases. Lastovicka and Thamodaran (1991) used exploratory factor analysis, analyzed correlations with maximum likelihood estimation, and considered other simulation parameters than those in this article, which may in part explain the differing results. We also conclude that standard errors are, in large, acceptably recovered in ordinary FSR. Problematic standard error estimates were found mainly in situations with low mean loading levels and highly correlated factors, which should not reflect the majority of practical settings. Thus, the conclusion by Skrondal and Laake (2001) that standard errors are in general “way off target” could not be supported. Differences in results may partly be due to the analysis of latent outcomes, scaling of factor variances with the reference-variable approach, and consideration of different simulation parameters in Skrondal and Laake (2001) compared with this article.

### Logistic Factor Score Regression

We conclude that the regression method is superior in logistic FSR. The regression method generally had the least problematic coefficient biases and empirical Type I error rates. Furthermore, standard errors were mostly estimated with an acceptable level of bias. However, practitioners should be aware that estimated relationships are in most cases negatively biased to a problematic extent in logistic FSR.

### Poisson Factor Score Regression

The simulations indicate that the regression method has the best overall performance in Poisson FSR. Given sufficiently large samples and high mean loading levels, the regression method was in many cases uniquely associated with acceptable coefficient bias levels. Nonetheless, standard errors were always severely underestimated. Therefore, significance testing should not be trusted in Poisson FSR even when coefficient biases are negligible.

In summary, the regression method is recommended for factor scoring in GLFSR. Still, the correlation-preserving method can be a viable alternative when factors are positively correlated and manifest variables have strong correlations on average with factors. In this case, one may choose to either maximize correlations between estimated and theoretical factor scores with the regression method or preserve interfactor correlations with the correlation-preserving method. However, we emphasize that the correlation-preserving method should not be used when factors are negatively correlated because of severe coefficient biases.

In almost all cases, the sum score methods produced severe coefficient biases that translated into empirical Type I error rates of 100%. Thus, despite their simplicity, this article strongly argues against the use of sum score methods in GLFSR, including the common special case with normally distributed outcomes.

Similar to Devlieger et al. (2016), the proportion of nonconvergent replications was the same for all factor scoring methods and regression models. In addition, we can conclude that nonconvergence issues are uniquely attributed to the measurement model estimation in GLFSR. Nonconvergence issues were eliminated if one analyzed either a large sample or a factor structure with a relatively high mean loading level. Because nonconvergent replications occurred mostly in situations with small samples and weak manifest variables, these cases should be interpreted with additional care.

In contrast to the majority of previous research, both equal and unequal loading coefficients were considered in this article. An interesting finding was that all previously discussed results held true in both homogeneous and heterogeneous loading structures with regard to the negligible impact of loading volatility on the performance criteria. From the perspective of external validity, we advocate the consideration of unequal loadings to reflect a broader range of loading structures found in practice.

Many aspects of GLFSR are still to be explored. For example, it would be of interest to examine how the results in this article may be influenced by different extents and types of missing data, model misspecification, choosing the reference-variable approach to scaling factors, and analyzing a latent outcome variable that can be either continuous or discrete. Results may differ from our simulation study depending on which scoring method is used for scoring the latent outcome variable and how scoring methods for independent and dependent latent variables are combined, which should be explored in future research. Moreover, it would be of interest to compare GLFSR with recent developments in the structural equation modeling literature to account for manifest and latent variables from exponential dispersion family distributions. However, generalized structural equation modeling is at the moment only available in a limited number of statistical software and with some model and output restrictions still to be addressed, rendering such comparisons difficult to make. In addition, it should be beneficial for practitioners if approaches to potentially offset problematic coefficient and standard error biases in GLFSR were investigated.

## Notes

1. The Bartlett method has intentionally been excluded. When only independent variables are latent, Devlieger et al. (2016) have demonstrated that Bartlett factor score estimates produce asymptotically biased FSR coefficient estimates, which is not the case for regression score estimates. It should be noted that the authors derived this result using a reference-variable approach to defining factor variance scales.

2. The bias-correcting FSR approaches by Croon (2002) and Hoshino and Bentler (2013), which allow for unbiased estimation of FSR coefficients, have not been included in this article. These methods require the estimator of the regression coefficients to have a closed-form expression that can be bias-corrected. Estimates are mostly found by iterative algorithms in generalized linear FSR because estimators generally have no closed-form expression. Therefore, the aforementioned bias-correcting methods have not been examined in our simulations. We see the development of bias-correcting FSR approaches that are broadly applicable in a generalized linear FSR context as an important future research topic.

3. It holds that Cov(X,F)=Corr(X,F)=ΛΦ given the measurement model setup. Recalling that factors have unit variances and cross-loadings are not allowed, each loading coefficient must be restricted to the interval [−1,1] and equal the correlation coefficient between the given factor and manifest variable.

4. It should be noted that the regression model can be specified with, for example, interaction terms and additional covariates. The model is kept as simple as possible in this article for the sake of clarity.

5. See, for example, Cox and Hinkley (1974) and Casella and Berger (2002).

6. The endpoints a and b satisfy $(a+b)/2=\mu_{\lambda }$ and $(b-a)/(2\sqrt{3})=\sigma_{\lambda }$ , b>a . $\mu_{\lambda }$ and $\sigma_{\lambda }$ are the mean and standard deviation of the uniform distribution.

7. Available from the authors on request.
